# The dynamic rotation axis of ulnohumeral joint during active flexion-extension: an in vivo 4-dimensional computed tomography analysis

**DOI:** 10.1186/s12891-022-05102-5

**Published:** 2022-02-16

**Authors:** Hua Liu, Erica Kholinne, Yucheng Sun, Tingting Liu, Jun Tan

**Affiliations:** 1 Department of Hand Surgery, Hand Surgery Research Center, Affiliated Hospital of Nantong University, College of Medicine, University of Nantong, 20 West Temple Road, Nantong, 226001 Jiangsu China; 2 Department of Orthopedic Surgery, Faculty of Medicine, Universitas Trisakti, St. Carolus Hospital, Jakarta, Indonesia; 3 Department of Medical Imaging, Affiliated Hospital of Nantong University, College of Medicine, University of Nantong, Nantong, Jiangsu China

**Keywords:** Four-dimensional computed tomography, Instantaneous rotation axis, Collateral ligament reconstruction, Elbow joint

## Abstract

**Background:**

As the collateral ligament reconstruction becomes more common to perform, the knowledge between the collateral ligament reconstruction and the elbow rotation axis is still ambiguous. The purpose of this study was to investigate the location of the intersections between the elbow rotation axis and medial and lateral aspect of the humerus.

**Methods:**

Four-dimensional computed tomography (4D CT) scan was designed to obtain the images from 8 participants. The instantaneous rotation axis was created according to the trochlea notch of the ulna in the Rapidform XO software. Then the intersections between the instantaneous rotation axis and the medial and lateral aspect of the humerus were identified in the Geomagic Wrap software. Landmark coordinate systems of the distal humerus was created.

**Result:**

The intersections in the medial aspect of the humerus were mostly located in the superior and posterior quadrant and showed the trend from anterior-superior to posterior-superior with the increment of the elbow flexion. The intersections in the lateral aspect of the humerus were mostly located in the middle half of the anterior quadrant and showed the trend from posterior-inferior to anterior-superior with the increment of the elbow flexion.

**Conclusion:**

There’s no isometric point for medial collateral ligament (MCL) and lateral ulnar collateral ligament (LUCL) reconstruction. The isometric area for MCL reconstruction should be considered at the superior and posterior quadrant of the medial aspect of the humerus. The isometric area for LUCL reconstruction should be considered at the middle half of the anterior quadrant of the lateral aspect of the humerus.

**Trial registration:**

This work was supported by the National Natural Science Foundation of China [No.81911540488] in 07/01/2019.

**Supplementary Information:**

The online version contains supplementary material available at 10.1186/s12891-022-05102-5.

## Background

The focus to seek for the elbow rotation axis grew parallelly with the need of improving the surgical outcome for elbow collateral ligament reconstruction. Current studies focused on the length changes of the MCL and LUCL between the ulnar and humerus footprint to find the isometric point in the bone, which was based on the theory that the rotation axis of the ulnohumeral joint was close to the axis connecting the center of the medial and lateral aspect of the distal humerus [3, 5, 18, 24]. The incorrect determination of the rotation axis of the elbow during extension-flexion motion may result in non-isometric ligament repairs, inappropriate external fixation of the elbow and incorrect elbow prosthesis design [6, 8].

Studies have been performed to investigate the axis of the ulnohumeral joint [6, 14, 15, 19], which was intended to improve the procedure of normal elbow joint restoration, i.e. the elbow collateral ligament reconstruction. The rotation axis of the ulnohumeral joint was defined with a fixed axis which passed through the center of the capitellum and trochlea without the consideration of the elbow extension - flexion [12, 23]. Two decades later, Duck et al. investigated the impact of the forearm position and mode of loading (passive/active) to define the elbow flexion axis in a cadaver setting [14]. Regardless, the previous studies were conducted in cadaver setting thus disregarding the physiologic upper extremity muscle force loading involvement which subsequently overcame by studies involving healthy subjects [6, 7, 12, 23]. Ericson et al. reported an intraindividual variation of elbow flexion axis of healthy participants which was located close to a line joining the center of trochlea and capitellum [15]. Similarly, in an in-vivo study using magnetic resonance imaging (MRI) by Goto et al. described that the averaged axis of rotation on the lateral condyle showed a circular pattern based on 3 forearm positions [19]. Later on, using a 4-positioned CT scans in healthy subjects, Adikrishna et al. found that the ulnohumeral joint was with a unique helical motion axis during extension–flexion [1]. It has become evident that the inconsistent elbow rotation axis resulted from previous studies were contributed by the limitation of experimental setting such as the lack of dynamic and real-time measurement in healthy subjects.

Four-dimensional computed tomography (4D CT) scan is a novel imaging technique, which has been used previously in wrist joint studies [11, 27, 29]. It enables the dynamic observation of the joint while maintaining the integrity of muscle force. This technique has shown potential for the assessment of active wrist motion and evaluation for the carpal instability. However, there’s no application in the elbow yet. The objective of this study was to identify the dynamic rotation axis of the elbow joint in healthy subjects and investigate the location of the intersections between the elbow rotation axis and medial and lateral aspect of the humerus. We hypothesized that the dynamic rotation axis was located around the center of the medial and lateral aspect of the humerus.

## Methods

### Study participants

After Institutional Review Board approval was obtained (No. 81911540488) and the statement about the design of the study within the principles of the Helsinki declaration was told, participants were enrolled to the study according to the inclusion and exclusion criteria. Inclusion criteria were (1) Age: 20–50 years old, (2) No previous history of collagen disease, (3) no evident trauma or deformity of the upper extremity, (4) consented for CT scan of the upper extremity. Exclusion criteria were (1) Restriction to elbow joint movement, (2) Prior evidence of elbow joint degeneration and deformity. Following this, 8 participants (3 males and 5 females) with the mean age of 26.6 years (range, 25 to 36) were enrolled to the study.

### Image acquisition and four-dimensional CT model reconstruction

**Fig. 1:**
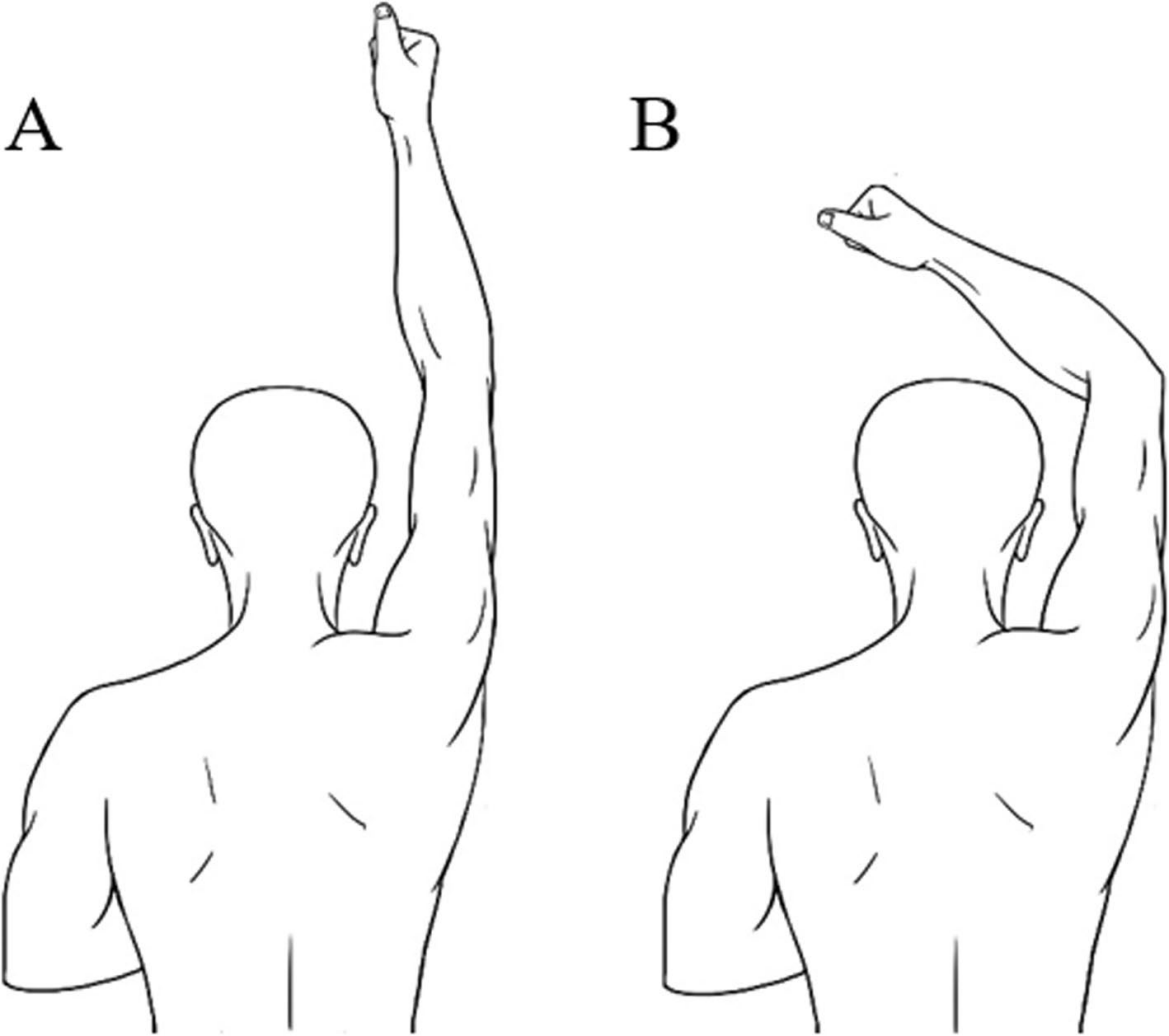
The postures when volunteers accepting the CT scan. The volunteers lied down in prone posture with their hand overhead and the wrist in neutral position (**A**). During the CT scanning, volunteers moved their forearms with the elbow joint static (**B**). (This figure was depicted by first author and was created by photoshop)

**Fig. 2:**
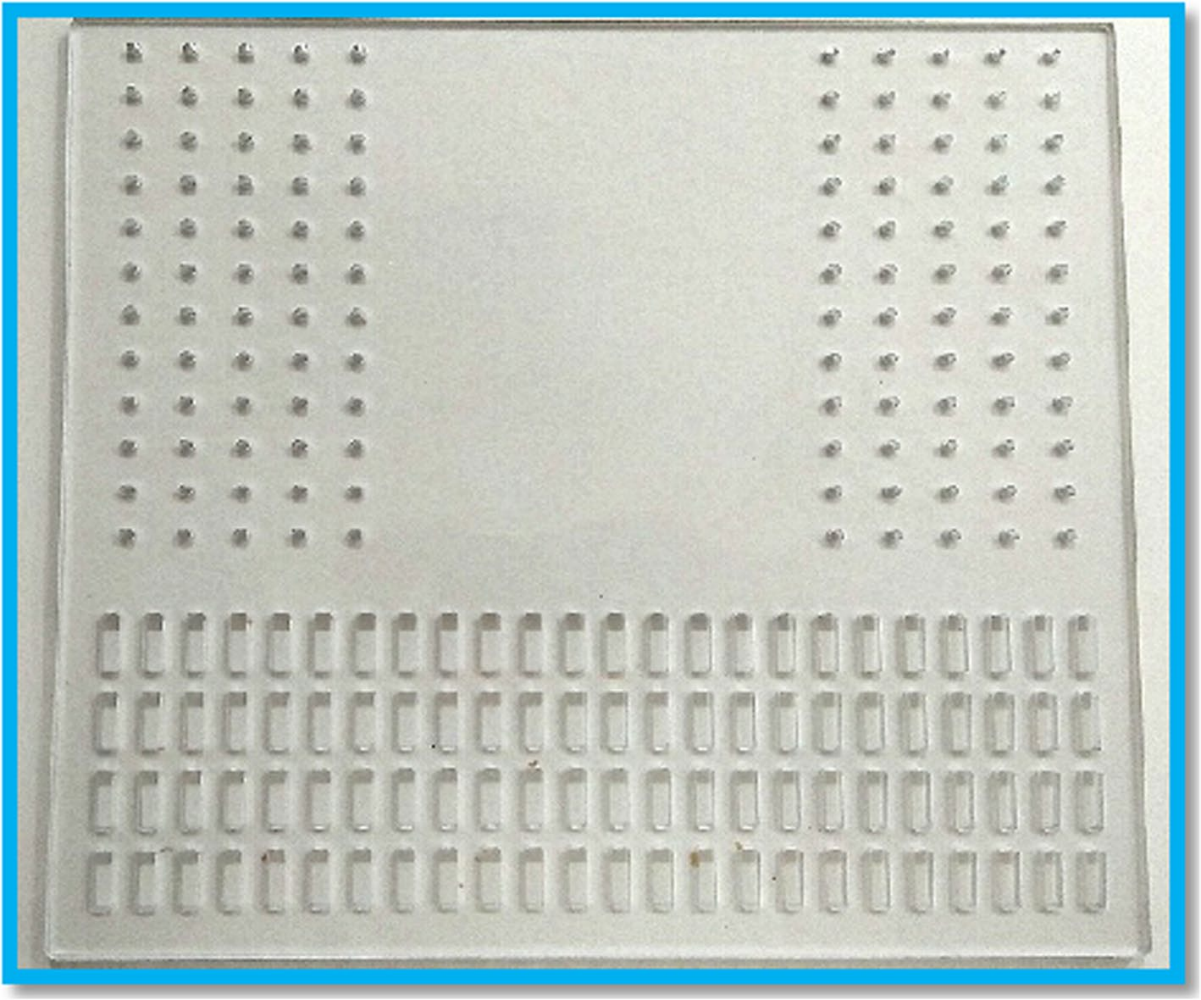
The board which was customized to fixed humerus during forearm motion. The baffles which were matched with the groove in the board were placed on the medial and lateral aspect of the humerus. (This figure was taken by first author’s mobile phone)

The distal humerus and the proximal radius and ulna were scanned using a CT scan (Revolution, GE Healthcare, Milwaukee WI, USA) with the protocol as follow: 256 row, 80 kVp, 200 mAs, 0.625 mm thickness, 1 s/circle. The participants lied in prone posture with hand above the head (Fig. 1). A custom-made board was used to fix the distal humerus during CT scanning (Fig. 2). Subsequently, participants were asked to move the elbow from maximum extension to the maximum flexion with the forearm and wrist maintained in neutral position during the 30 s of scanning time. Under this setting, a number of images were retrieved per one second of scanning time for each participant and one elbow position can be achieved for one second. The data was saved as DICOM (Digital Imaging and Communication in Medicine) files format.

### Image analysis

#### Determination of the rotational axis

MIMICS (Mimics Research 17.0, Materialise, Leuven, Belgium) software was used to convert scans into patient-specific 3D computer models. Single threshold-based methods were applied for the humerus, radius and ulna bone segmentation [28]. A single plane which was parallel to the whole coronal plane of the proximal ulna was determined. Similar steps were applied to the distal humerus bone to generate another plane. The angles between the ulnar and humeral planes were defined as the flexion angle.

**Fig. 3:**
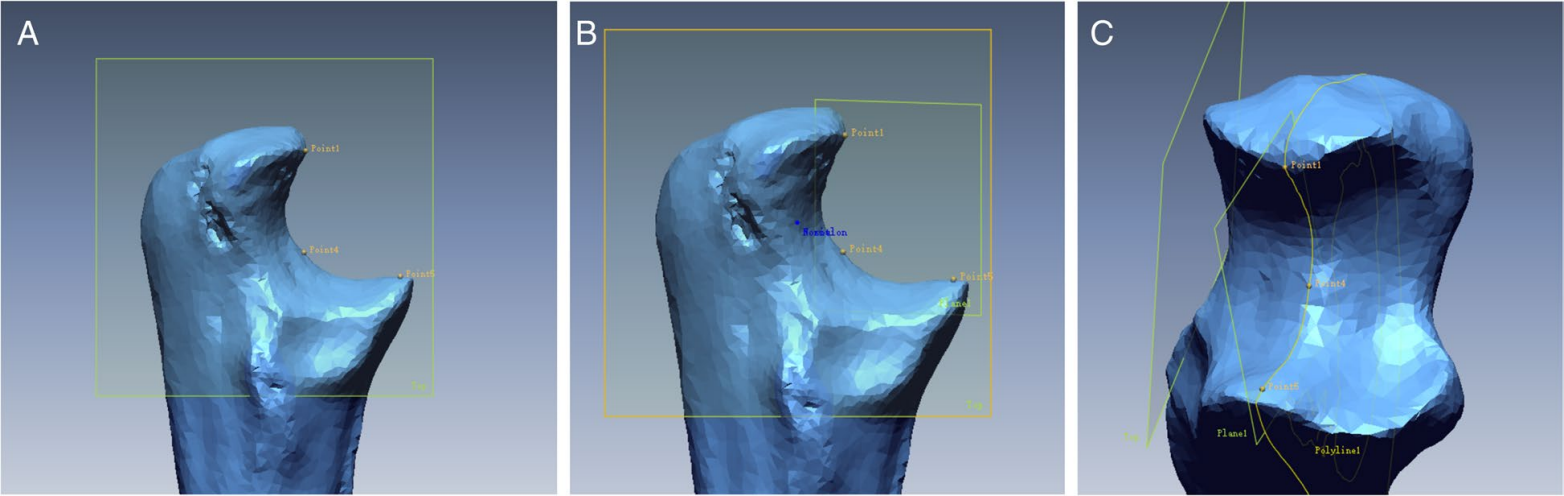
The process of the generation of the instantaneous rotation axis. Three dots which were located in the highest depression were registered and the plane which went through three dots was brought out (Fig. 3**A**). A curve went through the ulna surface and fitted the polyline which was the intersection of the plane and the surface of the ulna (Fig. 3**B**). The instantaneous rotation axis was generated by connecting the center of 11 curves (Fig. 3**C**)

The 3D reconstructed model was imported into 3D data processing software (Rapidform XO, JMR Systems, Derry, New Hampshire). A plane was generated at the bone surface by intersecting 3 registered dots at the greater sigmoid notch and was brought to intersect the outer surface of the greater sigmoid to generate a curve (Fig. 3A-B). A 1 mm-offset was created along the mediolateral projection to generate 11 curves. The center of the each best fitting line were determined to create 11 points to represent a line which defined as the instantaneous rotation axis (Fig. 3C) [20].

#### Determination of the landmark coordinate system

**Fig. 4:**
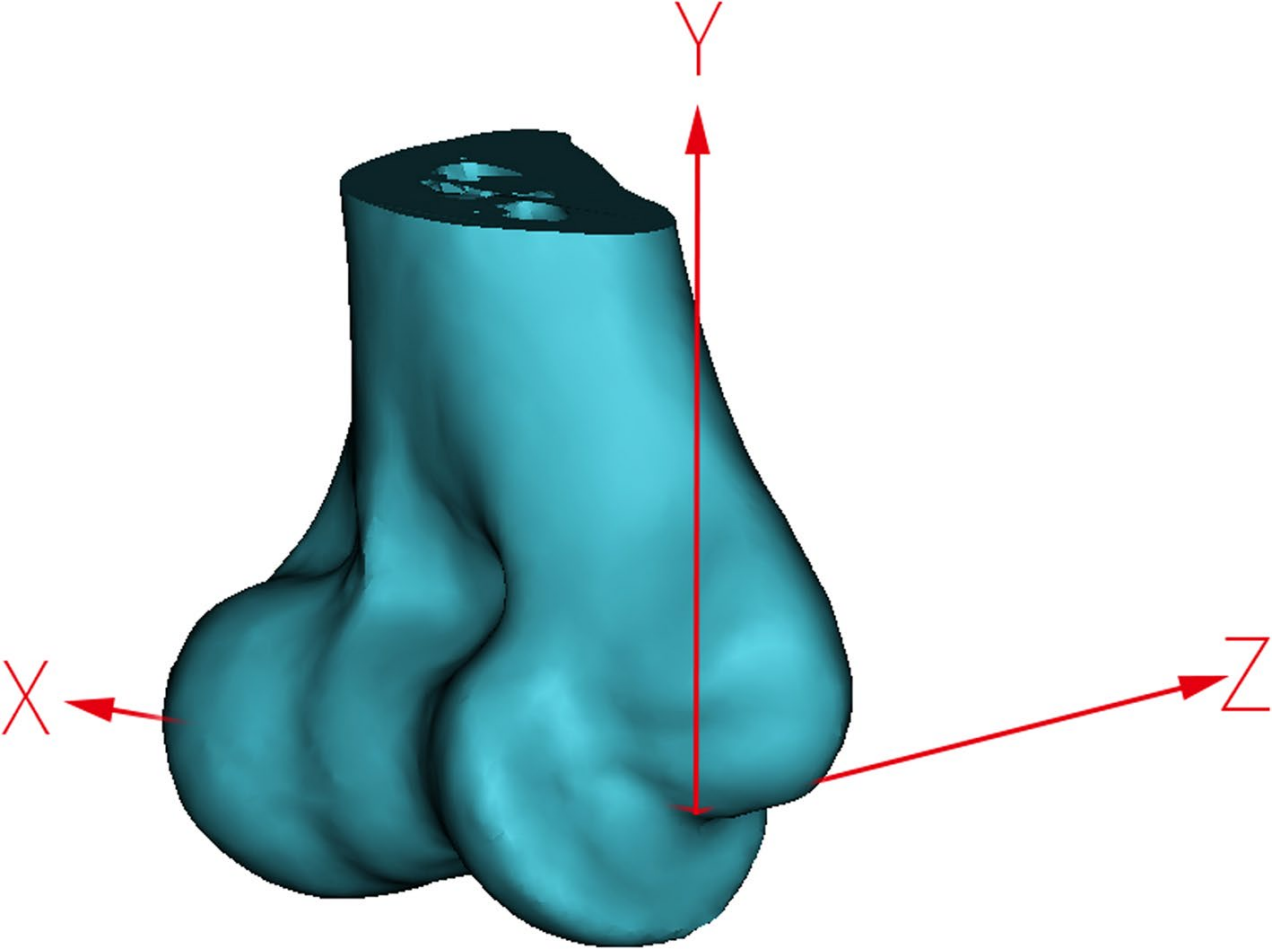
The coordinate system of the humerus. The original point is the projection of the center of the medial aspect humerus. X axis: connecting the centers of the medial and lateral aspect of the humerus toward laterally. Y axis: parallel to the longitudinal axis of the humerus toward superiorly. Z axis: perpendicular to the x- and z-axes toward posteriorly

Once the rotation axis was created, the landmark coordinate system and the intersections were created in the reverse engineering software (Geomagic Wrap 2015; Raindrop Geomagic, Durham, North Carolina). The X, Y, Z coordinate systems were defined (Fig. 4). The original point was defined as the projection in the medial epicondyle of the center of the medial aspect. The X axis represented the transcondylar axis. The Z axis was perpendicular to the X axis and humeral shaft, and Z axis toward the posterior side. The Y axis was perpendicular to the X axis and Z axis.

**Fig. 5:**
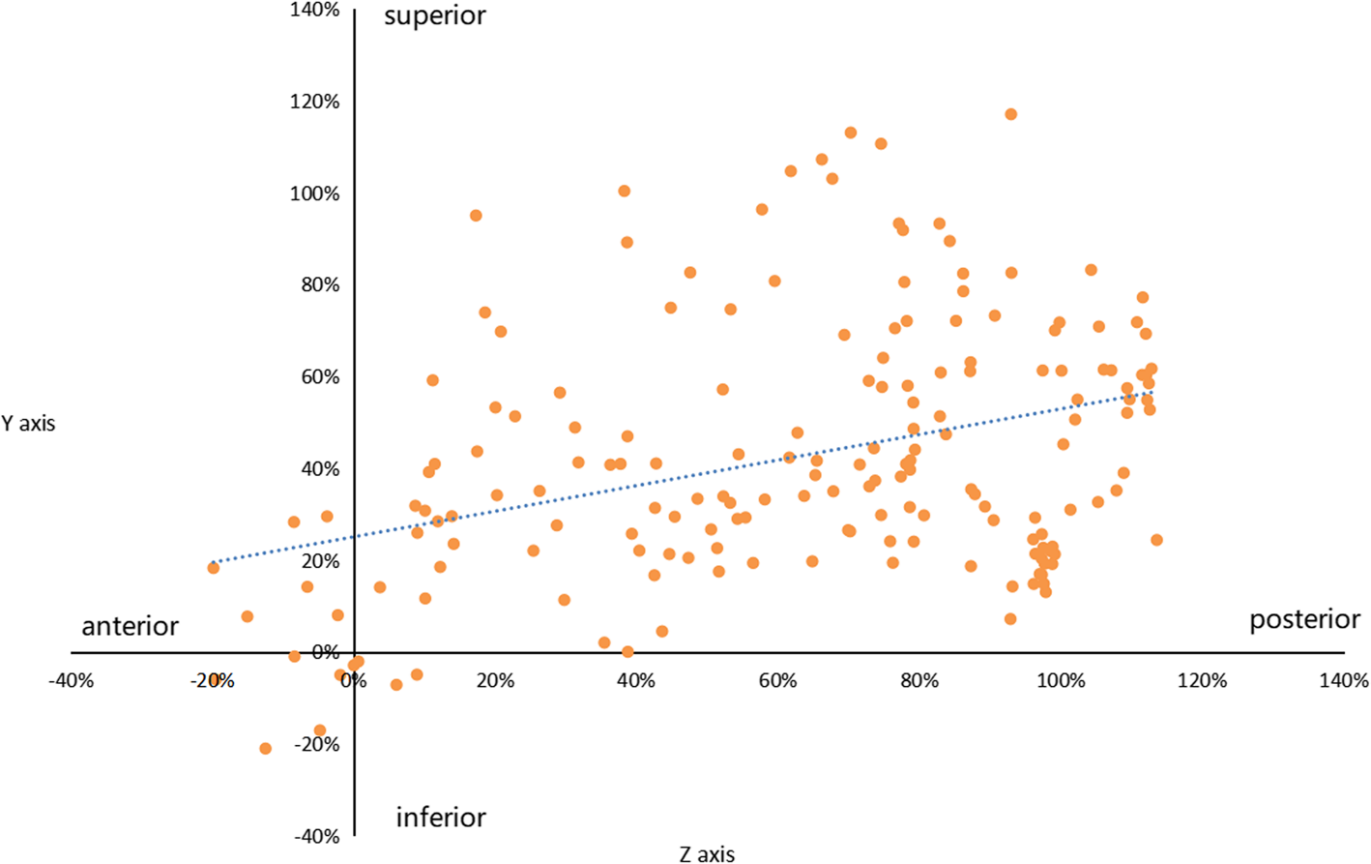
Progression of the intersections in the medial side of the humerus. The value of the horizontal axis represented the percentage which was calculated by the value of the z axis of the intersection divided by the radius of the medial aspect. The value of the longitudinal axis represented the percentage which was calculated by the value of the y axis of the intersection divided by the radius of the medial aspect. The original point was the projection of the center of the medial aspect humerus. The orange dots in the figure were the intersections between the rotation axis of the ulnohumeral joint and the medial aspect of the humerus. The blue dotted line represented the trend of the intersections from anterior-inferior to posterior-superior with the increment of the elbow flexion

**Fig. 6:**
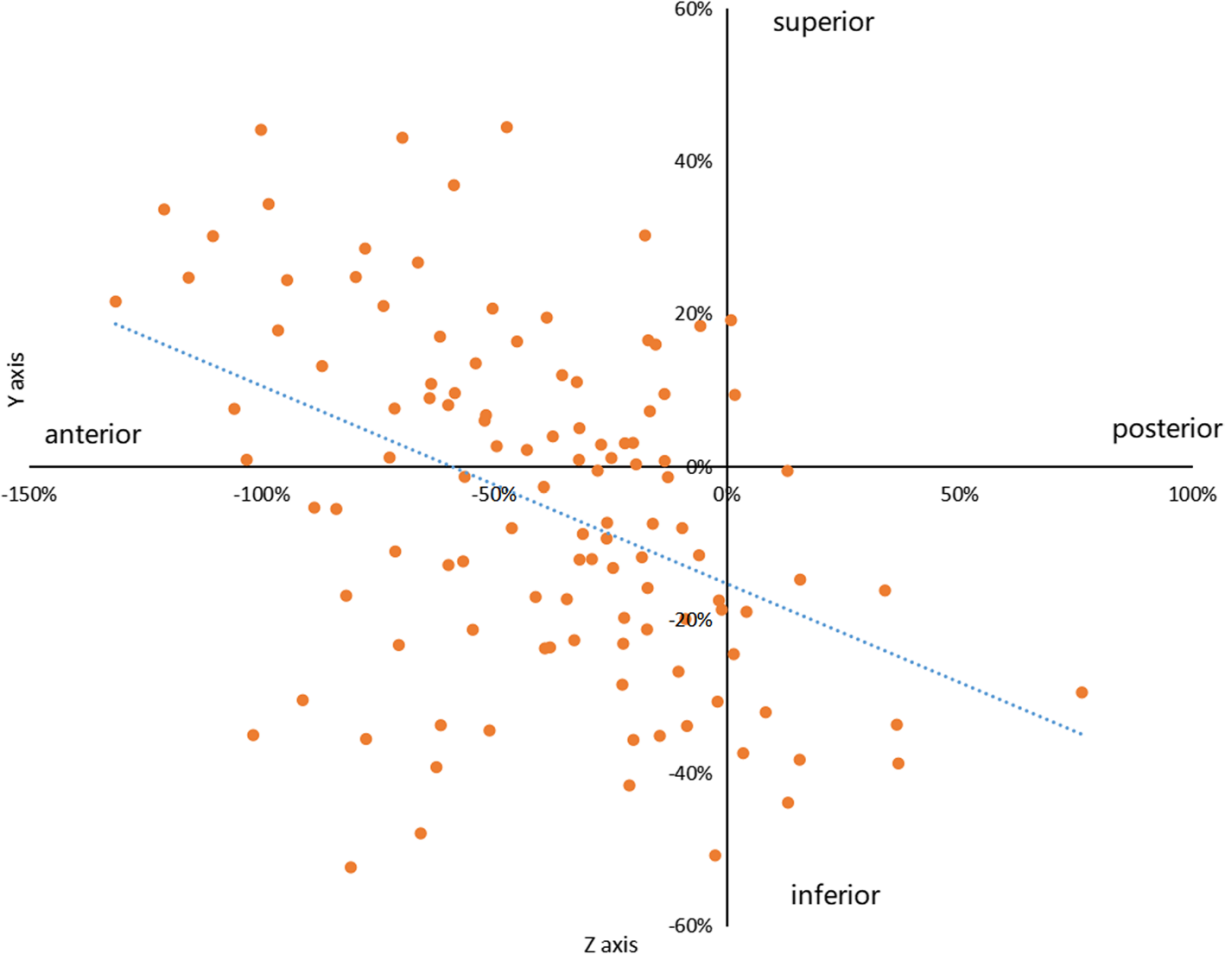
Progression of the intersections in the lateral side of the humerus. The value of the horizontal axis represented the percentage which was calculated by the value of the z axis of the intersection divided by the radius of the lateral aspect. The value of the longitudinal axis represented the percentage which was calculated by the value of the y axis of the intersection divided by the radius of the lateral aspect. The original point was the projection of the center of the medial aspect of the humerus. The orange dots in the figure were the intersections between the rotation axis of the ulnohumeral joint and the lateral aspect of the humerus. The blue dotted line represented the trend of the intersections from posterior-inferior to anterior-superior with the increment of the elbow flexion

All the intersections from all the participants were merged in one interposed figure (Fig. 5 and Fig. 6). Data normalization was performed using the value of the coordinate system divided by the radius of each lateral and medial aspect of the humerus.

### Statistical analysis

A power analysis was made to calculate the sample size. According to the value of the rotation axis calculated by Bottlang et al. [6], α was set as 0.05, power as 0.8, the value of group A as 2.6°, Group B as 5.7°and standard deviation as 2.2°, then they were brought in the formula of multi-sample mean comparison to estimate the sample size as 8. The coordinate values of the intersections in both lateral and medial aspect of the humerus were recorded. Also, the variation of the rotation axis in the horizontal plane and coronal plane was calculated. All data which was expressed as mean (SD) imported into SPSS 22.0 (SPSS Inc., Chicago, IL, USA). The data was analyzed with the linear regression. The significance level was set at 0.05.

## Results

### Instantaneous rotation axis

During the CT scan, the mean range of motion (ROM) was 106.5° (SD 3.8°). The mean variation between the instantaneous rotation axis and the X axis in the coronal plane and horizontal plane was 12.3° (SD 2.3°) and 45.5° (SD 14.8°), respectively. The intraindividual variation in the rotation axis ranging from 9.9 ° to 15.9° in the coronal plane and from 34.2° to 64.6° in the horizontal plane. With the flexion angle growing, the angle between the instantaneous rotation axis and the X axis in the horizontal plane increased at the same time (Video 1, *P* < 0.05). In the coronal plane, the trend of the instantaneous axis was inconsistent in each participant.

### Changes of the intersections in medial aspect of the distal humerus

The intersections in the medial aspect from all participants are shown in Fig. 5. The values of the Z axis and Y axis of the intersections were mostly exceeded 0 value which were located at the superior and posterior quadrant of the medial aspect of the distal humerus. With the flexion angle growing, the value of Z axis increased(*P* < 0.05). With the value of Z axis growing, the value of Y axis increased(*P* < 0.05). To illustrate, the intersections of the instantaneous rotation axis shifted from anterior-inferior to the posterior-superior quadrant at the medial aspect of the humerus in respect with the increment of the elbow flexion. Ranges of ±20% of the Y axis and Z axis was set as conditions simultaneously to obtain the symmetrical distribution of the intersections around the original point in the medial side, the corresponding flexion angle was between maximum extension and 30°.

### Changes of the intersections in lateral aspect of the distal humerus

The combined intersections in the lateral aspect from all participants are shown in Fig. 6. The values of the Z axis were mostly less than 0 value which were located at middle half of the anterior quadrant of the lateral aspect of the distal humerus. With the flexion angle growing, the value of Z axis decreased(*P* < 0.05). With the value of Z axis going down, the value of Y axis increased(*P* < 0.05). To illustrate, the intersections of the instantaneous rotation axis shifted from posterior-inferior to anterior-superior quadrant at the lateral aspect of the humerus in respect with the increment of the elbow flexion. Ranges of ±20% of the Y axis and Z axis were limited to the value of the Y axis and Z axis in the lateral side. 8 axes were picked out who went through ±20% of the Y axis and Z axis both in the medial and lateral side. The flexion angle of 8 axes were between maximum extension and 30°.

## Discussion

We investigated the instantaneous rotation axis of the elbow joint in normal subjects during extension-flexion motion with wrist in neutral position by 4D CT. The major finding of the current study was that the mean variation between the instantaneous rotation axis and the X axis in the coronal plane and horizontal plane is 12.3° and 45.5°, respectively. The intersections in the medial aspect of the humerus were mostly located in the superior and posterior quadrant and showed the trend from anterior-inferior to posterior-superior with the increment of the elbow flexion. The intersections in the lateral aspect of the humerus were mostly located in the middle half of the anterior quadrant and showed the trend from posterior-inferior to anterior-superior with the increment of the elbow flexion. The isometric point in the humerus for collateral ligament reconstruction was changing during the elbow extension-flexion motion.

The instantaneous rotation axis was shown to be inconsistent at the coronal plane. On the contrary, there was a variation of instantaneous rotation axis at the horizontal plane which corresponded to the increment of elbow flexion angle. When the elbow was put no more than 30°, the instantaneous rotation axis was close to the center of the capitulum and trochlea. Box-loop ligament reconstruction of the elbow was raised up to treat ligament tears both in MCL and LUCL [4, 17]. The attachment chosen in the medial aspect of the humerus was the origin point of the MCL, which was located in the anterior-inferior epicondyle. The attachment chosen in the lateral aspect of the humerus was the center of the capitulum. But the previous study by Finkbone et al. did not specify the flexion angle of the elbow joint when doing all collateral ligament reconstruction [17]. The distal humeral tunnel was created near the X axis which we defined in our study. Our study found the instantaneous rotation axis was shown to be close to the X axis when elbow flexion angle was less than 30°. For this reason, we recommended that during box-loop ligament reconstruction, distal humeral tunnel should be created while maintaining elbow in less than 30° position.

We also found that the intraindividual variation in the rotation axis ranging from 9.9 ° to 15.9° in the coronal plane and from 34.2° to 64.6° in the horizontal plane which was greater compared with the previous study by Ericson et al. [15]. Ericson et al. reported that the rotation axis was located close to a line joining the center of the trochlea and capitellum and intraindividual variation of the axis ranged from 2.1° to 14.3° in the coronal plane and 1.6° to 9.8° in the horizontal plane. The inconsistent result between Ericson et al. and the current study may be resulted from the different experiment setting which used plain x-ray and was taken at full extension and at 30°, 60°, 90° and 120° of flexion (static imaging) instead of the 4D CT scan with 30 positions used in the current study which represented the dynamic analysis. Since Duck et al. reported the impact of active/passive motion and forearm pronation/supination to the screw displacement axis [14], we speculated that another reason for the different result between our study and Ericson’s study was wrist position. The experiment in the current study was performed at neutral wrist position which represented an anatomic position in contrast with the supinated hand position performed in Ericson’s study.

The optimal method for MCL reconstruction has not yet been defined. Despite many techniques proposed, most of the technique emphasized on the importance of an appropriate graft attachment sites with the aim to achieve isometric ligament reconstruction as to restore normal elbow kinematics [5, 22], which was on the base of the theory that the rotation axis of the ulnohumeral joint passed through the centers of the capitellum and trochlea. Traditionally, the sublime tubercle served as the ulnar footprint of the reconstructed neo-ligament [13, 30]. However, quantitative analysis showed that the anatomic attachment of the MCL ulnar footprint was located more distal, namely the median ulnar ridge [16]. Also, there were different viewpoints about the location of the axis of the ulnohumeral joint [14, 15, 19]. For reasons above, the anatomical reconstruction may not serve as an isometric reconstruction because of the inconsistency of information regarding anatomic footprint of the ulnar site and different recognition of the axis of the ulnohumeral joint. Additionally, the MCL distal humeral footprint was widely accepted at the anteroinferior region of the distal humerus [2, 13, 30], which was regarded as the isometric point during the elbow motion. However, our study found that the axis of the ulnohumeral joint was not near the line connecting the centers of the medial and lateral aspect of the humerus and the isometric point in the humerus during the extension-flexion mode of the elbow joint was shifted from anterior-inferior to posterior-superior and mostly located at the posterior-superior quadrant of the medial epicondyle enface.

To the current knowledge, it is unclear to what position does the elbow joint need to be maintained during collateral ligament reconstruction. Patel et al. reviewed the outcomes and complications for the MCL reconstruction in 0° to 30° and 45° to 70° of the elbow joint, he came to the conclusion that the elbow flexion may not influence the return to the same or higher level of competition but appeared to influence the need for a revision after MCL reconstruction [26]. 0° to 30° flexion degree would result in a high revision rate. In our study, with the elbow moving from maximum extension to maximum flexion with elbow in neutral position, the intersection between the axis of the elbow and medial aspect of the humerus started near the anatomical attachment of the MCL in humerus and showed the trend from anterior-inferior to posterior-superior quadrant of the medial aspect of the humerus. For this reason, 0° to 30° flexion degree was recommended for the MCL reconstruction, which was also recommended by Cohen et al. because the reconstructions fixated at 30° more closely resembled the biomechanical characteristics of the intact elbow than did reconstructions fixated at 90° [9]. Current surgical techniques regarding the LUCL reconstruction were Morrey’s original tunnel technique and the contemporary docking technique [10]. The elbow was placed in 30° to 40° of flexion and forced pronation in docking technique and was placed in 30° of flexion and forced fully pronation in Morrey’s technique [21, 25]. The humeral tunnel was determined as the isometric point in the surgery, which was close to the anatomical footprint of the LUCL in the humerus. In our study, 30° of flexion was closer to the anatomical footprint of the LUCL compared with 30° to 40° of flexion since the isometric point in the lateral side was started near the anatomical attachment of the LUCL and showed the trend from posterior-inferior to anterior.

There has been an inconsistency report regarding isometric LUCL reconstruction [3, 18, 24]. Moritomo et al. found the most isometric point of the LUCL was located at the 2 mm proximal to the center of the capitellum in vivo MRI study [24]. Goren et al. reported that most isometric point on the humerus was located between the 3:00 and 4:30 o’clock positions on the lateral epicondyle in cadaveric biomechanical study [18]. Alaia et al. reported that the humeral center of rotation was the most isometric point for the humeral reconstruction site [3]. We postulated that the inconsistency of the reports was due to the different experiment setting. The current study found that there was no fixed isometric point during elbow motion and the points were started near the anatomical attachment of the LUCL and showed the trend from posterior-inferior to anterior-superior quadrant of the lateral aspect of the humerus. The isometric area for the LUCL reconstruction was located at the middle half of the anterior part of the lateral aspect of the humerus which was supported by Goren et al. [18].

Angle between the rotation axis and transcondylar line is 1.8°(SD6.3°) in maximum extension and 47.3°(SD13.9°) in maximum flexion. With the flexion angle grows, the intersections shifted from anterior-inferior to posterior-superior in the medial side and from posterior-inferior to anterior-superior in the lateral side. In other words, the axis showed the trend from posterior-superior in the medial side to anterior-superior in the lateral side in sagittal plane with the flexion angle grows.

There are several limitations to this study. Firstly, the flexion mode during CT scanning may not represent flexion in normal daily activity because wrist was naturally at the supination position when elbow is flexed. Secondly, the registration of the greater sigmoid notch of the ulna was performed manually. Thirdly, the flexion angle was not as large as the normal maximum flexion angle owing to the posture of the participant. Fourthly, the length of the reconstructed ligament and the translation along the axis were not evaluated.

## Conclusion

There’s no isometric point for medial collateral ligament (MCL) and lateral ulnar collateral ligament (LUCL) reconstruction. The isometric area for MCL reconstruction should be considered at the superior and posterior quadrant of the medial aspect of the humerus. The isometric area for LUCL reconstruction should be considered at the middle half of the anterior quadrant of the lateral aspect of the humerus.

## Supplementary Information

**Additional file 1.**

## Data Availability

The datasets used and/or analysed during the current study are available from the corresponding author on reasonable request.

## Abbreviations

4D CT: four-dimensional computed tomography
MCL: medial collateral ligament
LUCL: lateral ulnar collateral ligament
DICOM: Digital Imaging and Communication in Medicine
ROM: range of motion
